# A Systematic Intelligent Optimization Framework for a Sustained-Release Formulation Design

**DOI:** 10.3390/pharmaceutics17111419

**Published:** 2025-11-01

**Authors:** Yuchao Qiao, Yijia Wu, Mengchen Han, Hao Ren, Yu Cui, Xuchun Wang, Yiming Lou, Chongqi Hao, Quan Feng, Lixia Qiu

**Affiliations:** 1 Department of Health Statistics, School of Public Health, Shanxi Medical University, Taiyuan 030001, China; 2 Shanxi Provincial Key Laboratory of Major Diseases Risk Assessment, Shanxi Medical University, Jinzhong 030600, China

**Keywords:** sustained-release formulation, intelligent optimization algorithm, multi-objective optimization, exterior penalty function, multi-criteria decision-making

## Abstract

**Objectives**: This study proposes a systematic strategy for optimizing sustained-release formulations using mixture experiments. **Methods**: Model variables were identified and screened via LASSO regression, Smoothly Clipped Absolute Deviation (SCAD), and Minimax Concave Penalty (MCP), leading to the construction of a quadratic inference function-based objective model. Using this model, three multi-objective optimization algorithms—NSGA-III, MOGWO, and NSWOA—were employed to generate a Pareto-optimal solution set. Solutions were further evaluated through the entropy weight method combined with TOPSIS to reduce subjective bias. **Results**: The MCP-screened model demonstrated strong fit (AIC = 19.8028, BIC = 45.2951) and suitability for optimization. Among the Pareto-optimal formulations, formulation 45, comprising HPMC K4M (38.42%), HPMC K100LV (13.51%), MgO (6.28%), lactose (17.07%), and anhydrous CaHPO_4_ (7.52%), exhibited superior performance, achieving cumulative release rates of 22.75%, 64.98%, and 100.23% at 2, 8, and 24 h, respectively. Compared with the original formulation, drug release was significantly improved across all time points. **Conclusions**: This integrated workflow effectively accounted for component interactions and repeated measurements, providing a robust and scientifically grounded approach for optimizing multi-component sustained-release formulations.

## 1. Introduction

Sustained-release formulations are drug delivery systems designed to release active compounds at a predetermined rate, thereby maintaining stable plasma concentrations over an extended period to enhance therapeutic efficacy and reduce dosing frequency [1,2,3]. Such formulations are of great clinical importance in the long-term treatment of chronic diseases characterized by short drug half-lives, including cardiovascular disorders, diabetes, and central nervous system diseases [4,5,6]. However, existing studies still face limitations in modeling and optimization. Conventional full polynomial models fail to adequately capture complex component interactions, and the exponential growth of model variables with the increase in formulation components often leads to data non-saturation and variable redundancy. Moreover, most studies have not sufficiently accounted for the time dependence of cumulative release profiles, resulting in incomplete characterization of drug release kinetics. Single-objective transformation methods used in formulation optimization also rely heavily on subjective weighting, making it difficult to achieve balanced trade-offs among multiple objectives. Therefore, integrating mathematical modeling with intelligent optimization algorithms for multi-objective coordination has emerged as a promising research direction. Previous studies on lidocaine microemulsions and polycaprolactone microspheres experimentally demonstrated that combining mathematical modeling with multi-objective optimization can improve formulation development efficiency and predictive performance [7,8]. Thus, employing complex mathematical models in sustained-release formulation development is essential for systematically capturing component interactions, accounting for temporal release patterns, and enabling rational multi-objective optimization, ultimately facilitating the design of robust and high-performance formulations.

In this study, the monitored response variables were the cumulative drug release rates at 2 h, 8 h, and 24 h (Y_2_, Y_8_, and Y_24_), representing the initial, intermediate, and sustained-release stages, respectively. These responses provide a direct assessment of the formulation’s release performance over time and serve as the primary objectives for subsequent multi-objective optimization. To achieve efficient modeling, it is essential to address the problem of data non-saturation arising from high-dimensional variables in mixture design. Traditional full polynomial models are inherently limited as, the number of generated variables increases exponentially with the number of formulation components, leading to insufficient sample size and increased experimental burden. The q-Component Centered Polynomial (q-CCP) method refines polynomial terms within a (q − 1)-dimensional simplex while maintaining model symmetry, thereby reducing experimental cost and improving predictive accuracy [9,10,11]. When the number of variables far exceeds the sample size, feature selection becomes crucial. Least Absolute Shrinkage and Selection Operator (LASSO) achieves variable selection through coefficient shrinkage but may introduce bias in highly correlated settings [12,13]. Smoothly Clipped Absolute Deviation (SCAD) reduces small-coefficient estimation bias via non-convex penalties while satisfying the oracle property [14,15], and the Minimax Concave Penalty (MCP) mitigates local optimum risks by tuning penalty concavity, making it suitable for non-saturated data [14]. These approaches enable the identification of key interaction effects and improve the robustness and predictive accuracy of sustained-release models.

After variable selection, capturing time dependence becomes critical for building robust statistical models. The cumulative release rate of sustained-release systems typically exhibits repeated measurements and temporal correlation. The Quadratic Inference Function (QIF) provides improved estimation efficiency and robustness over Generalized Estimating Equations (GEE) as, it does not rely on strict assumptions regarding the correlation matrix and achieves greater computational stability and variance consistency, particularly under limited sample conditions or with unknown correlation structures [16,17]. The QIF has been widely applied in pharmacokinetic and longitudinal biomedical analyses, maintaining high estimation efficiency and predictive accuracy even in datasets with strong temporal dependence or small sample sizes [18,19]. Incorporating the QIF into sustained-release modeling allows for more precise characterization of component interactions and time-dependent effects, offering a statistically robust foundation for subsequent multi-objective optimization.

Formulation optimization represents a critical step in enhancing the performance of sustained-release systems. Traditional methods such as goal programming and contour plotting typically convert multi-objective problems into single-objective ones through weight assignment or target transformation; however, these approaches are highly dependent on subjective weighting and fail to capture trade-offs among objectives comprehensively [20]. In contrast, intelligent multi-objective optimization algorithms possess strong global search capabilities, enabling simultaneous balancing of multiple objectives while generating diverse Pareto-optimal solutions and reducing the likelihood of local optima [21]. Recently developed algorithms—including the Non-Dominated Sorting Genetic Algorithm III (NSGA-III), the Multi-Objective Grey Wolf Optimizer (MOGWO), and the Non-Dominated Sorting Whale Optimization Algorithm (NSWOA)—have demonstrated high efficiency, robustness, and rapid convergence in complex optimization tasks such as path planning, scheduling, and resource allocation [22,23]. Compared with traditional genetic algorithms and single-objective optimization strategies, these methods are particularly suitable for high-dimensional multi-objective problems as, they preserve solution diversity and uniformity while providing more stable and comprehensive optimization outcomes. Therefore, applying intelligent multi-objective optimization algorithms to sustained-release formulation design holds significant potential, especially for complex multi-objective decision scenarios.

In summary, this study establishes a systematic methodological framework for modeling and optimizing sustained-release formulations. The proposed framework employs the q-CCP method for variable generation; integrates LASSO, SCAD, and the MCP for key variable selection and interaction identification; utilizes the QIF for time-dependent modeling; and finally implements multi-objective global optimization through NSGA-III, MOGWO, and NSWOA. This framework provides a generalized mathematical and decision-support platform for sustained-release formulation design and process optimization, enhancing model robustness, predictive precision, and overall development efficiency.

## 2. Materials and Methods

### 2.1. Materials

This study was based on experimental data of glipizide–hydroxypropyl-β-cyclodextrin sustained-release tablets reported in the literature [24] for modeling and optimization. The original formulation, designed using a D-optimal mixture design, included five key excipients—HPMC K4M (X_1_), HPMC K100LV (X_2_), MgO (X_3_), lactose (X_4_), and anhydrous CaHPO_4_ (X_5_)—which were treated as study factors in the modeling analysis. According to the original study, all components were passed through an 80-mesh sieve, weighed according to the designed proportions and mixed using the equal-volume incremental method. The cumulative drug release rate was used as the formulation evaluation metric to assess drug release behavior under different pH conditions: the 2 h cumulative release in pH 1.2 medium (Y_2_) reflected the initial release, while the 8 h (Y_8_) and 24 h (Y_24_) cumulative release in pH 6.8 medium represented sustained-release performance and release completeness. Using this dataset, formulation variable screening, interaction analysis, and multi-objective optimization were performed without actual tablet preparation. The composition and dosage ranges of the formulations are listed in Supplementary Table S1.

According to the quality requirements for glipizide-related preparations specified in the Chinese Pharmacopoeia, the acceptable ranges for Y_2_, Y_8_, and Y_24_ are 15–25%, 55–65%, and 80–110%, respectively, with 110% set as the upper limit for complete dissolution. Based on preliminary formulation research results, the formulation components and their dosage ranges were further determined (shown in Supplementary Material Table S1), serving as the foundation for formulation optimization studies. The experimental protocol and results for the glipizide sustained-release tablets are presented in Table 1.

### 2.2. Methods

#### 2.2.1. Feature Selection Methods

Regularization-based variable selection techniques are widely used for high-dimensional modeling in pharmaceutical formulation studies. Among them, LASSO, proposed by Tibshirani in 1996, introduces an L1 penalty into least squares regression to simultaneously perform coefficient estimation and variable selection, achieving sparsity and interpretability while avoiding the high computational burden of traditional subset selection methods [13]. However, LASSO applies uniform shrinkage to all coefficients, which may cause “group selection bias” when variables are highly correlated, and its performance is sensitive to the penalty parameter determined via cross-validation [14]. Fan and Li developed SCAD in 2001 to address these limitations. SCAD employs a non-convex penalty that strongly shrinks small coefficients while limiting shrinkage on large coefficients, mitigating estimation bias and improving stability [15]. It possesses Oracle-like properties and handles correlated variables more effectively than LASSO, though the non-convex optimization can result in local optima and higher computational cost. The MCP, proposed by Zhang in 2010, further reduces estimation bias while preserving sparsity by gradually decreasing the penalty on large coefficients through a concavity parameter γ [25]. Compared with SCAD, the MCP has a simpler functional form, faster convergence, and greater robustness in the case of non-saturated or high-dimensional data, though it still requires careful tuning of γ.

Overall, LASSO, SCAD, and the MCP represent a progressive evolution of regularization-based variable selection methods, balancing sparsity, stability, and computational efficiency. In this study, these three methods were independently applied to the q-CCP-based high-dimensional feature set to screen formulation and interaction variables significantly associated with the cumulative drug release behavior of glipizide sustained-release tablets. We aimed to evaluate the respective effectiveness and suitability of each method for formulation modeling by comparing the selected variables and predictive performance. Table 2 summarizes the main characteristics of the models and differences between them, providing a concise reference for selecting an appropriate method in complex formulation optimization.

#### 2.2.2. Quadratic Inference Function

GEE is a classical method for modeling repeated measurements or longitudinally correlated data introduced by Liang & Zeger (1986) that estimates parameters based on a specified working correlation matrix [26]. However, the accuracy and efficiency of GEE depend strongly on correct correlation specification; misspecification can lead to biased estimates and reduced efficiency, particularly with complex correlation structures or limited sample sizes. The QIF, proposed by Qu et al. in 2000, extends GEE by representing the inverse of the correlation matrix as a linear combination of basis matrices [18], which addresses these shortcomings. This reformulation eliminates the need for explicit estimation of complex correlation parameters and enhances robustness in case of model misspecification. The quadratic construction of the QIF further enables the direct evaluation of model goodness-of-fit, facilitating model selection and comparison.

Compared with GEE, the QIF provides several distinct advantages: (i) it yields consistent estimates even when the working correlation matrix is misspecified, thereby reducing model bias; (ii) it naturally integrates model fit assessment within its estimation framework; and (iii) it demonstrates higher efficiency in small-sample or complex correlation scenarios. Nevertheless, its performance can be sensitive to the selection of basis matrices, and computational demands may increase under high-dimensional or large-scale data conditions.

In the context of this study, the cumulative drug release data of glipizide sustained-release tablets represent longitudinal observations measured at multiple time points (2 h, 8 h, and 24 h), where successive measurements are correlated within each formulation. Here, the QIF was employed to model these intra-formulation correlations by estimating regression parameters that link formulation variables (excipient proportions and interaction terms) with the observed cumulative release rates. The correlation structure among repeated measurements was incorporated through basis matrices within the QIF framework, allowing more accurate estimation of time-dependent release characteristics and improving the interpretability and stability of the predictive model. Thus, the QIF provided a statistically robust approach for analyzing correlated release profiles and optimizing formulation performance. Table 3 summarizes the principal differences between GEE and the QIF.

#### 2.2.3. Exterior Penalty Function Method

In multi-objective optimization, it is often necessary to balance multiple conflicting objectives under a set of constraints. In the optimization of sustained-release formulations based on mixture design, an equality constraint arises from the requirement that the sum of all component proportions equals 1 (or 100%) [27,28] This constant-sum constraint ensures that the total content of excipients remains fixed, reflecting the physical and practical boundaries of the formulation system.

The exterior penalty function method is widely applied to address such constrained problems for its simplicity and flexibility. It introduces penalty terms into the objective function to account due to constraint violations, converting the constrained problem into an unconstrained one which is solvable via standard optimization algorithms. When a constraint is violated, a penalty proportional to the deviation is imposed, guiding the solution toward feasibility. The method’s performance depends on the penalty coefficient, a value that is too small may yield infeasible results, while an excessively large value can cause instability and slow convergence. Proper tuning of the penalty factor is therefore essential for both feasibility and computational efficiency [14,25,29].

In this study, the exterior penalty function was integrated into the multi-objective optimization framework to manage the equality constraint inherent in the mixture design ($\sum X_{i}=82.8\%$). This ensured that the total proportion of the five excipients—HPMC K4M, HPMC K100LV, MgO, lactose, and anhydrous CaHPO_4_—remained constant during optimization. The objectives were defined according to the cumulative release rates at 2 h, 8 h, and 24 h (Y_2_, Y_8_, and Y_24_), representing the initial, intermediate, and terminal phases of drug release. The penalty term maintained compositional feasibility while allowing intelligent optimization algorithms to identify Pareto-optimal solutions with balanced release performance across time points, ensuring both mathematical validity and physical interpretability in sustained-release formulation design.

#### 2.2.4. Multi-Objective Optimization Algorithm

Three representative intelligent optimization algorithms were employed in this study to address the multi-objective conflicts and complex constraints inherent in sustained-release formulation optimization—NSGA-III, MOGWO, and NSWOA. These algorithms improve convergence efficiency while maintaining the diversity and uniformity of Pareto-optimal solutions, enabling comprehensive exploration of the solution space.

NSGA-III, proposed by Deb et al. (2014), is an advanced extension of NSGA-II specifically developed for high-dimensional multi-objective problems [30,31]. It introduces a reference-point mechanism and adaptive normalization strategy to achieve uniform solution distribution along the Pareto front, effectively balancing convergence and diversity during population evolution. MOGWO, developed by Mirjalili et al. (2015), extends the Grey Wolf Optimizer to handle multi-objective problems by mimicking the social hierarchy and cooperative hunting strategies of grey wolves [32,33]. It employs a leader-based search structure (α, β, and δ wolves) and an external archive to preserve non-dominated solutions, enhancing convergence speed, robustness, and Pareto front uniformity in nonlinear optimization scenarios. NSWOA, an improved version of the Whale Optimization Algorithm by Mirjalili and Lewis (2016), integrates non-dominated sorting and crowding distance mechanisms to extend WOA’s capability to multi-objective optimization [34]. NSWOA is inspired by the foraging behavior of humpback whales—encircling prey, bubble-net feeding, and random search—and achieves uniform Pareto front coverage and balanced global exploration [35].

In this study, these three algorithms were applied to the same multi-objective optimization framework, where the decision variables were the proportions of five excipients (HPMC K4M, HPMC K100LV, MgO, lactose, and anhydrous CaHPO_4_) under the equality constraint $\sum X_{i}=82.8\%$. The objective functions were defined using the cumulative release rates at 2 h (Y_2_), 8 h (Y_8_), and 24 h (Y_24_), representing the initial, intermediate, and sustained-release stages, respectively. By comparing their convergence behavior, distribution characteristics, and optimization performance, we evaluated the effectiveness of each algorithm in identifying Pareto-optimal formulation designs that balance early burst control, release stability, and overall dissolution completeness.

#### 2.2.5. Multi-Objective Optimization Evaluation

In multi-objective optimization, a large number of Pareto-optimal solutions are typically generated, often exhibiting considerable variability across objectives. When multiple and potentially conflicting criteria are involved, selecting a single, most desirable solution becomes a key challenge as improvement in one objective often results in deterioration of others [36]. The Entropy Weight Method (EWM) and the Technique for Order of Preference by Similarity to Ideal Solution (TOPSIS) were combined in this study for post-optimization evaluation to achieve a rational and quantitative selection of the optimal sustained-release formulation. The optimization objectives were the cumulative release rates at 2 h, 8 h, and 24 h (Y_2_, Y_8_, and Y_24_), which collectively describe the release performance of the sustained-release system.

The EWM was first applied to determine the objective weights of these release indicators based on their information entropy, ensuring that the weighting process reflected the intrinsic variability of each parameter without subjective bias [37]. The normalized decision matrix X was constructed from the Pareto solutions, and the probability distribution P_ij_ and entropy values e_j_ were calculated to derive the weight vector. These weights were then incorporated into the TOPSIS method, which evaluates and ranks Pareto-optimal formulations according to their Euclidean distance from the positive and negative ideal solutions [38].

#### 2.2.6. Function Transformation

The experimental data in this study must satisfy the constant-sum constraint of the mixture design which means the sum of all mixture components is a fixed value (*X*_1_ + *X*_2_ + *X*_3_ + *X*_4_ + *X*_5_ = 82.8%). The exterior penalty function method was accordingly used to transform the objective function optimization problem with equality constraints into an unconstrained extremum problem. The initial penalty factor *σ* was set to 1000 by constructing an unconstrained extremum-seeking function:

$$Q=\hat{Y}+\sigma P\left(x\right)$$

where $P\left(x\right)=\sum_{j=1}^{l}\varphi \left(h_{j}\left(x\right)\right)$ denotes the penalty term, *h*_*j*_(*x*) is the constraint function, and *φ* (⋅) is the penalty function. The corresponding transformations were implemented for the subobjective functions at different time points:

When t = 2 h, the transformed objective function was

$$Q_{1}=-{\hat{Y}}_{2}+\sigma P\left(x\right)$$

When t = 2 h, the transformed objective function was

$$Q_{2}=-{\hat{Y}}_{8}+\sigma P\left(x\right)$$

When t = 2 h, the transformed objective function was

$$Q_{3}=-{\hat{Y}}_{24}+\sigma P\left(x\right)$$

Through the conversion, the original constrained optimization problem was transformed into an unconstrained optimization problem, enabling the efficient determination of optimal solutions at each time point while ensuring that the fixed and constraint conditions of the mixture design were satisfied. This method provided a mathematical guarantee for the subsequent optimization of the model.

#### 2.2.7. Software and Parameter Settings

The LASSO, SCAD, and MCP methods from the ncvreg package in R version 4.3.3 were employed to screen variables generated with Scheffe center polynomial modeling. Optimization for all three methods was performed using the Optimization Toolbox in MATLAB R2022a. The NSGA-III parameter settings were as follows: single-point crossover probability P_c_ = 0.8, mutation probability P_m_ = 0.05. The MOGWO parameter settings were as follows: initial population size N = 100, grid expansion factor alpha = 0.1, leader selection pressure factor beta = 5, additional archived individual selection pressure gamma = 2. In the NSWOA parameter settings, the result output frequency during iteration was set to ishow = 10. The initial population size N was uniformly set to 100, and the number of evolutionary generations is set to 200. Each algorithm was independently run 15 times to ensure the reliability and reproducibility of results. Using the SPSS 26.0 software, the objective weights for each evaluation metric were calculated via the entropy method.

## 3. Results

### 3.1. Results of the Feature Selection

Using 10-fold cross-validation, three variable selection methods—LASSO, SCAD, and the MCP—were applied to screen characteristic variables from the mixture design sustained-release formulation trial data of glipizide pH-independent sustained-release tablets. LASSO, SCAD, and the MCP identified 16, 12, and 10 variables, respectively. The selected features and their regression coefficients for each method are presented in Supplementary Information Tables S2–S4. As evidenced by the above results, the MCP obtained fewer variables during variable selection than LASSO and SCAD, while its post-selection model demonstrated superior interpretability. Furthermore, the MCP method better accounted for interactions among variables during the selection process, enhancing the model’s predictive capability and stability. The MCP method consequently exhibited superior performance in this study, providing a more streamlined and effective feature variable set for subsequent model construction.

### 3.2. Results of the QIF Modeling

**Figure 1:**
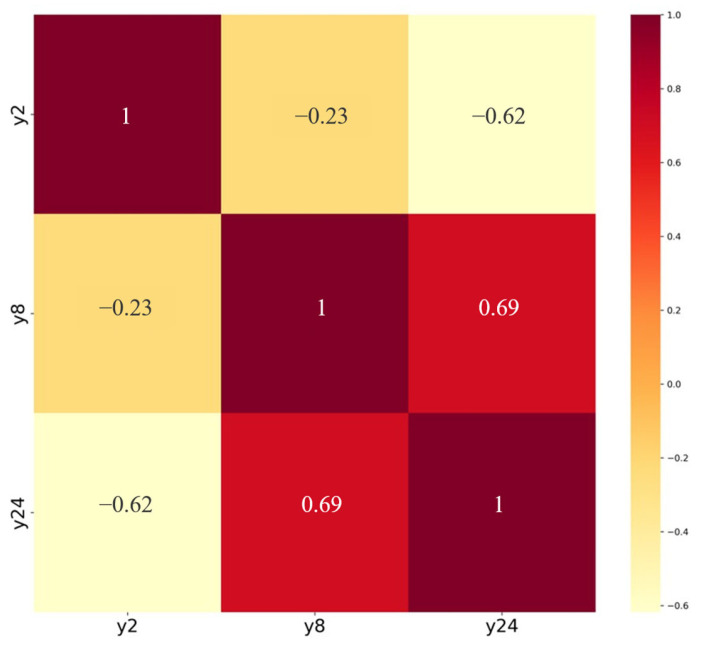
Heatmap of correlation coefficients for cumulative release levels at three time points.

A correlation analysis was conducted on the drug release levels of the sustained-release formulation at three time points, with results shown in Figure 1. The analysis revealed correlation coefficients of 0.23 between Y_2_ and Y_8_, 0.62 between Y_2_ and Y_24_, and 0.69 between Y_8_ and Y_24_. This indicated that measurements at one time point exhibited a certain degree of correlation with those at adjacent time points, demonstrating characteristics of autocorrelation. Therefore, an autocorrelation matrix structure was set to account for the data’s autocorrelation when establishing models using secondary inference functions, thereby better reflecting the dependencies among data points. This configuration contributed to enhancing the model’s fitting accuracy and stability.

To evaluate the impact of different variable selection methods on model fit, we modeled the data using three approaches: no variable selection, LASSO, SCAD, and the MCP. Model fit was assessed using AIC and BIC metrics. The results are presented in Supplementary Material Table S5. It is evident that models without variable selection exhibit relatively poor fit, as indicated by higher AIC and BIC values. In contrast, model fitting improved after variable selection using the LASSO, SCAD, and MCP methods. The model with variables selected via the MCP method performed best, yielding an AIC value of 19.8028 and a BIC value of 45.2951. Therefore, the Scheffe-centered polynomial model based on the MCP method demonstrated the best model fit. This model was ultimately selected as the final modeling result for this study. The specific model establishment results are shown in Table 4.

Based on the results in Table S5 of Supplementary Information, the QIF model was established after variable selection using the MCP method for Scheffe’s central polynomials. The model was expressed as follows:

$$\begin{array}{ll} & \hat{Y}=-4.27112-0.01113X_{2}X_{4}-0.00974X_{2}X_{5}-0.61399X_{3}X_{5}-0.00165X_{1}X_{3}X_{4}\\ & +0.01076X_{1}X_{3}X_{5}+0.00005X_{1}X_{2}X_{3}X_{4}X_{5}+0.02473X_{3}t-0.00049X_{5}t-0.21985t^{2}\\ & +9.08641t \end{array}$$

By substituting different time points (t = 2, 8, and 24 h) into the above model, sub-objective function models corresponding to each release stage were established. The detailed mathematical formulations of these sub-objective functions are provided in the Supplementary Information. Based on this model, the cumulative drug release at different time points can be quantitatively predicted, allowing for comprehensive analysis of the effects of formulation variables on release behavior. This modeling framework effectively captures both the interaction among components and the temporal dynamics of drug release, thereby offering a reliable quantitative basis for subsequent multi-objective formulation optimization.

### 3.3. Results of the Multi-Objective Algorithm Optimization

*Q*_1_, *Q*_2_, and *Q*_3_ were identified as target functions requiring optimization to achieve optimal drug release at different time points in this study. Three intelligent optimization algorithms were employed to conduct optimization searches within the search space. Each multi-objective optimization method underwent 15 random searches, ultimately yielding a set of superior and relatively reasonable optimization solutions. During the optimization process, the maximum fitness evolution curve is used to evaluate the algorithm’s convergence performance. It reflects the trend of changes in the best solution the algorithm can find during iterations and serves as a key indicator for determining whether the algorithm can approach the global optimum. The average fitness evolution curve, on the other hand, reflects the overall evolutionary level and dynamic performance of the population. It can be used to observe population diversity and the stability of the algorithm during the optimization process.

**Figure 2:**
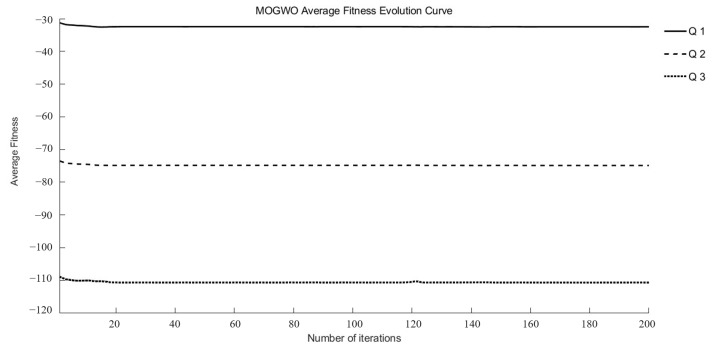
Evolutionary curves of average fitness for MOGWO.

**Figure 3:**
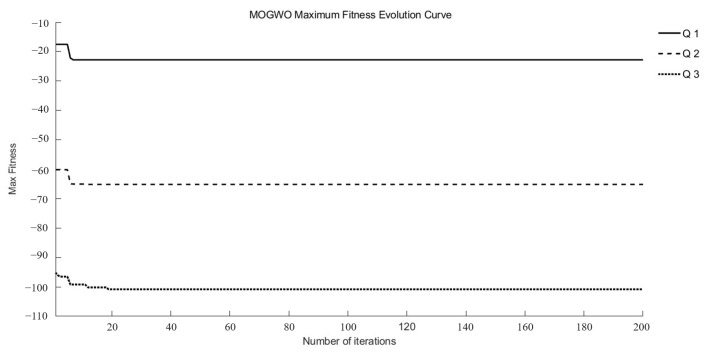
Evolutionary curves of maximum fitness for MOGWO.

As illustrated in Figure 2 and Figure 3, the MOGWO algorithm exhibited a rapid convergence trend, with both the mean and maximum fitness values stabilizing after approximately 6–12 generations. This indicates that the algorithm efficiently balanced exploration and exploitation during the optimization process, achieving satisfactory convergence stability. The convergence behaviors of NSGA-III and NSWOA, presented in Supplementary Information Figures S1–S4, followed similar patterns. Specifically, NSGA-III reached a stable convergence state between generations 7 and 10, while NSWOA demonstrated slightly faster stabilization between generations 5 and 9. These results collectively confirm that all three algorithms successfully achieved convergence within a limited number of iterations, ensuring the reliability and robustness of the optimization process.

As summarized in Table 5, the optimal solutions obtained by the three multi-objective optimization algorithms all satisfied the relevant drug release criteria under fixed component ratio constraints. Notably, NSGA-III and NSWOA exhibited superior convergence efficiency and solution stability, whereas MOGWO, despite converging at a slightly slower rate, was still capable of producing feasible and effective optimization outcomes.

As shown in Table 5, all three multi-objective optimization algorithms yielded optimal solutions meeting drug release standards while satisfying mixing ratios and constraints. To eliminate subjectivity from manual selection, the entropy weight method and TOPSIS method were employed in this study for comprehensive evaluation of the 45 optimized candidate solutions. The evaluation results effectively distinguished the relative merits of different solutions, ultimately identifying satisfactory Pareto-optimal solutions that provide scientifically viable references for optimizing drug preparation processes.

### 3.4. Results of the Entropy Weight-TOPIS Method Optimization Evaluation

The cumulative release degree was employed as an evaluation metric in this study given the potential for significant subjective bias in the traditional manual selection of optimal solutions. A comprehensive evaluation was conducted on the 45 optimized solutions by integrating a multi-objective assessment framework combining the entropy weight method and TOPSIS method. Through this evaluation approach, a set of satisfactory Pareto-optimal solutions was successfully identified, providing decision-makers with an objective, scientific, and practical drug preparation plan. The specific analysis results indicated that in a pH 6.8 medium, the weight assigned to the 8 h cumulative drug release rate (Y_8_) was the highest. Relevant data are shown in Supplementary Material Table S6. Based on the weight values of each evaluation indicator, the data were weighted and subjected to TOPSIS analysis. Since the cumulative release degree was a positive indicator, the value of the negative ideal solution was 0. A set of satisfactory Pareto-optimal solutions was ultimately selected by calculating the similarity (C value) between each evaluation object and the satisfactory solution scheme and then sorting them by C value. Partial results are shown in Table 6 (complete results are shown in Supplementary Information Table S8).

### 3.5. Comparison of Optimization Results

In this study, the q-CCP method was employed to generate modeling variables during the optimization process. An optimal modeling framework combining three variable selection methods based on coefficient compression was constructed to form the objective function. Prescription optimization was conducted using three multi-objective optimization algorithms, yielding multiple Pareto solution sets. Finally, an integrated assessment combining the entropy weight method and TOPSIS were performed to screen a set of objectively feasible and satisfactory Pareto non-dominated solutions. Compared to the original formulation, Solution 45 in the optimal solution set achieved significant improvements in cumulative drug release rates: a 1.85% increase in the 2 h cumulative release rate at pH 1.2, a 5.48% increase in the 8 h cumulative release rate at pH 6.8, and an 8.73% increase in the 24 h cumulative release rate. Correspondingly, the formulation composition of the drug preparation also underwent notable changes. The proportions of HPMC K100LV, MgO, and lactose increased by 35.13%, 25.50%, and 13.79%, while the proportions of HPMC K4M and anhydrous CaHPO_4_ decreased by 3.95% and 41.23%, respectively. This formulation possessed good rationality and optimization effects while satisfying the mixing ratio and constraint conditions. Specific optimization results are shown in Table 7. Additionally, Table 8 lists other schemes superior to the original scheme (e.g., Schemes 29, 15, and 44). Researchers can select the most suitable scheme from these preferred options based on specific experimental requirements and conditions.

## 4. Discussion

A novel integrated optimization framework for sustained-release formulation design was proposed and applied in this study. This method combines the q-CCP approach, regularization-based variable selection methods (LASSO, SCAD, and MCP), QIF modeling, multi-objective intelligent optimization algorithms (NSGA-III, MOGWO, and NSWOA), and the entropy weight–TOPSIS comprehensive evaluation approach. This framework enables the simultaneous characterization of high-order interactions and nonlinear effects in mixture designs, identifies key variables, and balances drug release performance with formulation feasibility during multi-objective optimization. Finally, a quantitative ranking of the Pareto-optimal solution set was achieved through the entropy weight–TOPSIS evaluation, allowing objective screening and decision support for optimized formulations, and providing a systematic pathway toward intelligent sustained-release formulation design [39,40].

In terms of optimization performance, the proposed framework achieved a notable improvement compared with the reference formulation reported in the literature. The reference formulation was derived using Design-Expert, which was used to perform statistical analysis and polynomial fitting of experimental data to generate ten candidate formulations within the specified composition range. The final formulation was selected based on the evaluation index and the maximum similarity factor (f_2_) [24]. In contrast, the optimized formulation predicted in this study showed increases in cumulative drug release of 1.85%, 5.48%, and 8.73% at 2 h, 8 h, and 24 h, respectively, indicating a potential enhancement in sustained-release performance. Furthermore, the multi-objective optimization process generated not only a single optimal solution but also a series of Pareto-optimal formulations satisfying the mixture constraints [41]. This provides researchers with greater flexibility to select appropriate formulations according to specific experimental requirements, process conditions, or development priorities. Based on model predictions, these candidate formulations notably demonstrated potential advantages in release behavior and formulation rationality, suggesting that the proposed optimization framework may exhibit good robustness and practical applicability [42].

From a pharmaceutical mechanistic perspective, the adjustment of excipient proportions in the optimized formulation was highly consistent with the known mechanisms of sustained-release matrix tablets. An increased amount of HPMC K100LV enhanced gel layer formation and system viscosity, prolonging the diffusion path and leading to smoother drug release [43]. A higher MgO content regulated the microenvironmental pH within the tablet, improving the stability and dissolution of weakly acidic drugs in acidic media [44]. An increased proportion of lactose contributed to the formation of a porous matrix structure, enhancing liquid penetration and diffusivity [43]. Meanwhile, a moderate reduction in HPMC K4M and anhydrous dibasic calcium phosphate content prevented excessive gel compaction, maintaining tablet integrity and an appropriate release rate [45]. These compositional adjustments align well with the release kinetics of sustained-release formulations, indicating that the optimization results are not only numerically reasonable but also pharmaceutically mechanistic and theoretically sound.

A high-order q-CCP model was employed in this study at the methodological and statistical modeling levels to capture the complex nonlinear and high-order interaction effects among excipients [42,46]. Compared with traditional response surface and full factorial designs, the q-CCP model provides a more comprehensive characterization of nonlinear relationships in mixture designs, offering richer information for multivariate formulation systems [47]. By integrating the MCP variable selection method, model sparsity was achieved, allowing the effective retention of key variables and their interactions while suppressing redundant factors, thereby improving model interpretability. It is noteworthy that although the non-convex penalty structure of the MCP enhances the identification of significant variables [48], its performance may be influenced by factors such as sample size, variable correlation, and data distribution characteristics; therefore, the variable selection results should be interpreted in the context of specific data conditions. An autocorrelation matrix structure was incorporated in this study for the repeated-measures cumulative release data, and the QIF for model fitting. Compared with the traditional Generalized Estimating Equations approach, QIF provides more robust modeling of within-subject correlation structures, improving estimation efficiency for repeated-measures data and enhancing the reliability and interpretability of multi-time point release predictions [39,49]. This methodological foundation ensured strong statistical support for subsequent optimization procedures.

In the multi-objective optimization phase, the combined application of NSGA-III, MOGWO, and NSWOA effectively balanced global search capability and the diversity of Pareto-optimal solutions. NSGA-III maintained a uniform distribution of solutions in high-dimensional objective spaces and rapidly approached the Pareto front [50]; MOGWO exhibited strong local search and adaptive capabilities [51]; and NSWOA demonstrated the ability to explore potential solution sets under complex constraint conditions [52]. The integration of these three algorithms not only enhanced optimization space coverage but also reduced the local optimum bias inherent to single algorithms, thereby improving the diversity and representativeness of the Pareto solution set. Subsequently, the EWM combined with TOPSIS was used to comprehensively rank the Pareto-optimal solutions, enabling quantitative comparison of multi-indicator schemes that balance drug release performance and formulation feasibility while minimizing subjective bias in decision-making [53]. This approach provides an operable and quantitative basis for sustained-release formulation design under multi-constraint and multi-objective conditions.

Overall, a systematic optimization framework for sustained-release formulation design was established in this study by integrating high-order polynomial modeling, sparse variable selection, autocorrelation fitting, multi-objective intelligent optimization, and entropy-weighted TOPSIS evaluation. The proposed framework not only improved the model’s interpretability for complex nonlinear and interactive effects but also generated diverse and practical candidate solutions in the multi-objective optimization process, providing researchers with a rational basis for selecting optimal formulations under varying constraints. Moreover, the proposed methodology simultaneously considers drug release performance, formulation rationality, and process feasibility, offering quantitative guidance and a referential operational framework for the development and industrialization of sustained-release formulations.

## 5. Conclusions

Our study proposed an optimization workflow integrating the q-CCP approach with variable selection methods. The formulation parameters of sustained-release tablets were systematically optimized via the combination of the QIF and multi-objective optimization algorithms. Through comprehensive evaluation using the EWM and TOPSIS, a satisfactory Pareto non-dominated solution set was identified. Based on the modeling results derived from the experimental data reported by Fang et al., the optimized formulation exhibited improved predicted cumulative release performance compared with traditional methods, with simulated release rates at 2 h, 8 h, and 24 h increasing by 1.847%, 5.483%, and 8.727%, respectively. Although this study provides a systematic and scientific framework for formulation optimization in sustained-release drug delivery, future experimental validation will be necessary to confirm its practical applicability.

## 6. Limitations

Although our study introduced methodological innovations and proposed a comprehensive optimization process, certain limitations remain. First, while we obtained optimization results and demonstrated significant improvements, we did not conduct actual experimental validation. Consequently, the effectiveness and feasibility of the optimization scheme in practical applications cannot be confirmed. Subsequent studies should incorporate experimental validation to confirm the performance of the proposed optimization scheme in actual production. Meanwhile, our research primarily focused on enhancing drug release rates, while other drug properties such as stability and dissolution rates require further consideration. Future work may incorporate these factors into the optimization objectives to construct a more comprehensive optimization model.

## Figures and Tables

**Table 1:** Experimental protocol and results for glipizide pH-non-dependent sustained-release tablets.

Schemes	Pharmaceutical Components (%)					Cumulative Release Degree (%)		
	HPMC K4M (X_1_)	HPMC K100LV (X_2_)	MgO (X_3_)	Lactose (X_4_)	Anhydrous CaHPO_4_ (X_5_)	2 h (Y_2_)	8 h (Y_8_)	24 h (Y_24_)
1	29.5	22.6	7.2	16.4	7.1	17.1	64.4	98.1
2	25.2	27.8	2.0	22.8	5.0	17.5	54.4	87.3
3	41.4	10.0	10.0	16.4	5.0	16.0	59.8	102.3
4	38.3	14.4	4.5	17.8	7.8	19.3	61.2	93.2
5	32.5	17.5	5.0	22.8	5.0	17.7	58.8	92.5
6	29.0	27.8	6.0	15.0	5.0	19.2	69.8	100.4
7	45.4	10.0	2.0	20.4	5.0	14.9	64.2	95.0
8	25.0	26.3	4.8	18.4	8.4	13.7	63.8	100.8
9	25.0	25.0	10.0	17.8	5.0	19.3	62.5	90.6
10	45.4	10.0	2.0	15.0	10.4	23.2	60.2	89.0
11	32.5	17.5	10.0	15.0	7.8	17.4	59.6	93.9
12	34.0	18.7	4.3	17.8	7.8	15.3	59.8	103.0
13	25.2	27.8	2.0	15.0	12.8	19.6	55.1	83.8
14	40.0	10.0	5.0	15.0	12.8	26.3	60.5	91.2
15	25.2	27.8	2.0	22.8	5.0	20.3	58.2	89.0
16	29.5	21.8	3.2	20.3	7.9	23.8	64.4	94.7
17	25.2	27.8	2.0	15.0	12.8	20.8	55.3	84.5
18	47.8	13.0	2.0	15.0	5.0	18.2	65.1	95.3
19	45.4	10.0	2.0	20.4	5.0	16.3	63.8	99.5
20	32.5	17.5	2.0	18.0	12.8	16.0	61.9	98.7
21	41.4	10.0	10.0	16.4	5.0	16.7	58.8	87.4
22	40.4	20.4	2.0	15.0	5.0	20.1	60.7	87.4
23	33.0	27.8	2.0	15.0	5.0	19.7	56.0	86.5
24	40.0	10.0	2.0	22.8	8.0	19.0	56.7	91.2
25	40.0	10.0	5.0	15.0	12.8	22.5	58.4	90.6

**Table 2:** Comparative Overview of LASSO, SCAD, and MCP.

Feature/Criterion	LASSO	SCAD	MCP
Proposed by/Year	Tibshirani, 1996	Fan & Li, 2001	Zhang, 2010
Penalty Type	Convex (L1)	Non-convex	Non-convex
Main Idea	Adds L1 penalty to achieve coefficient shrinkage and variable selection	Strongly shrinks small coefficients, weakly penalizes large ones	Gradually reduces penalty for large coefficients via concavity parameter (γ)
Key Advantages	Simple, interpretable, efficient for high-dimensional data (*p* > *n*)	Oracle property, avoids over-shrinkage, handles correlated variables well	Balanced sparsity and unbiasedness, faster convergence, robust under non-saturated data
Main Limitations	Equal shrinkage causes group selection bias; sensitive to penalty parameter	May trap in local optima; high computational cost	Requires tuning of γ; may show instability under high correlation
Computation Complexity	Low	Moderate to High	Moderate
Typical Applications	Pharmaceutical modeling, gene screening, financial prediction	Pharmaceutical and epidemiological modeling	High-dimensional optimization, machine learning, formulation studies

**Table 3:** Comparison of QIF and GEE.

Feature/Criterion	GEE	QIF
Proposed by/Year	Liang & Zeger, 1986	Qu et al., 2000
Core Idea	Estimates parameters using specified working correlation matrix	Approximates inverse of correlation matrix via linear combination of basis matrices
Handling of Correlation	Relies on correct specification of working correlation matrix	Robust to misspecification of correlation structure
Efficiency	May be inefficient with small sample size or complex correlation	Generally more efficient under finite sample or complex correlation conditions
Goodness-of-Fit Assessment	Limited; requires additional methods	Naturally incorporates model fit tests via quadratic construction
Computational Complexity	Moderate	Higher for very large/high-dimensional data due to matrix operations
Typical Applications	Longitudinal and repeated measures studies	Repeated measures modeling, formulation optimization, complex longitudinal data

**Table 4:** Results of the second-order regression function modeling for glipizide pH-independent sustained-release tablets.

Parameters	Estimated Value	Standard Error	*Z*	*P*
Intercept	4.2711	<0.0001	6,839,553.34	<0.0001
*X*_24_	−0.0111	0.0002	−50.01	<0.0001
*X*_25_	−0.0097	0.0001	−145.17	<0.0001
*X*_35_	−0.6140	<0.0001	−194,734.95	<0.0001
*X*_134_	−0.0017	0.0004	−4.20	<0.0001
*X*_135_	0.0108	<0.0001	243.54	<0.0001
*X*_12345_	0.0001	<0.0001	12.97	<0.0001
*X*_3*t*_	0.0247	<0.0001	1488.84	<0.0001
*X*_5*t*_	−0.0005	<0.0001	−7.16	<0.0001
t*t	−0.2199	<0.0001	−7196.43	<0.0001
time	9.0864	<0.0001	2,702,798.87	<0.0001

t represents time, and t*t represents the square of time.

**Table 5:** Satisfactory solution sets for glipizide pH-independent sustained-release tablets optimized using three multi-objective optimization algorithms.

Optimization Methods	Schemes	Pharmaceutical Ingredients					Cumulative Release Degree		
		*X*_1_	*X*_2_	*X*_3_	*X*_4_	*X*_5_	*Y*_2_	*Y*_8_	*Y*_24_
NSGA-III	1	36.786	11.010	9.126	19.382	6.497	18.421	61.084	97.463
	2	33.465	20.442	2.953	20.694	5.247	19.610	61.360	95.307
	3	25.217	23.427	7.792	15.251	11.115	19.378	61.829	97.645
	4	30.039	21.690	3.386	20.997	6.690	20.154	61.965	96.071
	5	30.066	15.112	2.690	22.218	12.714	20.332	62.021	95.805
	6	25.199	18.156	6.158	21.725	11.563	20.638	62.845	98.010
	7	31.293	15.830	7.244	22.492	5.941	20.606	62.991	98.630
	8	27.350	17.315	9.844	20.670	7.622	20.312	63.078	99.733
	9	28.589	15.035	5.915	21.651	11.612	20.912	63.083	98.152
	10	34.957	12.257	4.536	20.992	10.060	21.492	63.463	97.999
	11	27.365	20.745	8.401	15.921	10.370	21.092	63.636	99.698
	12	31.423	18.737	4.185	16.744	11.714	22.102	64.016	98.399
	13	34.848	16.477	3.089	16.782	11.606	22.268	64.020	97.970
	14	26.544	22.193	4.914	20.584	8.566	22.127	64.159	98.856
	15	30.737	17.228	6.657	20.785	7.396	22.456	64.749	100.145
MOGWO	16	25.000	27.800	10.000	15.000	5.000	18.685	61.481	98.218
	17	26.845	16.251	9.996	22.169	7.539	18.940	61.729	98.444
	18	37.269	18.310	2.990	19.033	5.198	20.144	61.900	95.862
	19	35.501	17.066	2.656	20.937	6.642	20.396	62.098	95.916
	20	25.437	21.107	8.909	21.144	6.204	20.406	63.039	99.339
	21	30.350	25.226	3.143	16.339	7.743	20.871	62.643	96.645
	22	45.380	12.056	2.000	15.000	8.364	21.171	62.771	96.316
	23	42.505	11.207	2.000	15.241	11.847	21.210	62.799	96.317
	24	43.351	13.841	3.193	16.317	6.099	21.309	63.092	97.127
	25	41.335	17.011	2.685	15.009	6.761	21.458	63.164	96.993
	26	33.684	13.244	8.600	18.226	9.046	21.465	64.042	100.193
	27	27.207	23.936	5.577	19.627	6.453	21.618	63.754	98.729
	28	37.560	19.496	5.629	15.115	5.001	21.994	64.142	99.149
	29	34.682	19.649	8.314	15.000	5.156	22.160	64.703	100.765
	30	37.852	14.293	3.695	15.123	11.838	23.019	64.860	99.049
NSWOA	31	36.758	12.245	3.426	21.961	8.411	21.083	62.894	97.004
	32	37.177	14.354	5.786	16.485	9.004	21.186	63.346	98.384
	33	37.834	13.160	5.379	16.507	9.925	21.352	63.448	98.318
	34	37.957	13.310	5.828	16.589	9.121	21.475	63.640	98.694
	35	37.477	12.039	5.400	19.884	8.001	21.579	63.684	98.578
	36	37.765	13.067	5.553	16.845	9.574	21.797	63.920	98.862
	37	37.337	13.923	5.741	16.795	9.008	21.858	64.011	99.031
	38	38.468	12.654	5.799	16.442	9.440	22.337	64.497	99.537
	39	38.084	15.617	5.117	16.204	7.782	22.371	64.435	99.218
	40	37.991	13.178	5.806	16.710	9.119	22.384	64.546	99.591
	41	39.069	12.346	5.239	16.276	9.873	22.492	64.568	99.383
	42	38.803	12.318	5.419	16.442	9.821	22.498	64.600	99.487
	43	39.331	12.360	5.208	16.550	9.352	22.607	64.679	99.486
	44	38.198	13.213	5.766	16.474	9.152	22.741	64.898	99.927
	45	38.422	13.513	6.275	17.068	7.523	22.747	64.983	100.227

**Table 6:** Results of the TOPSIS.

Schemes	Optimal Ideal Solution Distance D+	Negative Ideal Solution Distance D−	Relative Proximity C	Sorting Results
45	0.009	0.129	0.932	1
29	0.013	0.128	0.908	2
15	0.014	0.123	0.901	3
44	0.014	0.125	0.898	4
43	0.022	0.117	0.839	5
40	0.023	0.115	0.834	6
42	0.023	0.115	0.831	7
38	0.024	0.113	0.823	8
41	0.025	0.114	0.819	9
30	0.028	0.118	0.809	10
39	0.029	0.109	0.79	11
26	0.03	0.109	0.782	12
28	0.035	0.102	0.747	13
14	0.038	0.101	0.728	14
37	0.038	0.098	0.72	15

**Table 7:** Comparison of optimization results.

Schemes	Pharmaceutical Ingredients					Cumulative Release Degree		
	*X*_1_	*X*_2_	*X*_3_	*X*_4_	*X*_5_	*Y*_2_	*Y*_8_	*Y*_24_
Original document optimal scheme	40.000	10.000	5.000	15.000	12.800	20.900	59.500	91.500
Scheme 45	38.422	13.513	6.275	17.068	7.523	22.747	64.983	100.227
Change amount	−1.578	3.513	1.275	2.068	−5.277	1.847	5.483	8.727
Rate of Change (%)	−3.945	35.130	25.500	13.787	−41.227	8.837	9.215	9.538

**Table 8:** Comparison of Satisfactory Schemes with Original Scheme.

Schemes	Pharmaceutical Ingredients					Cumulative Release Degree		
	*X*_1_	*X*_2_	*X*_3_	*X*_4_	*X*_5_	*Y*_2_	*Y*_8_	*Y*_24_
Original document optimal scheme	40.000	10.000	5.000	15.000	12.800	20.900	59.500	91.500
45	38.422	13.513	6.275	17.068	7.523	22.747	64.983	100.227
29	34.682	19.649	8.314	15.000	5.156	22.160	64.703	100.765
15	30.737	17.228	6.657	20.785	7.396	22.456	64.749	100.145
44	38.198	13.213	5.766	16.474	9.152	22.741	64.898	99.927

## Data Availability

All data generated or analyzed during this study are included in the article and its Supplementary Materials.

## Supplementary Materials

The following supporting information can be downloaded at: https://www.mdpi.com/article/10.3390/pharmaceutics17111419/s1, Table S1: Scope setting table for each component, Table S2: Lasso screening variables and regression coefficients, Table S3: SCAD screening variables and regression coefficients, Table S4: MCP screening variables and regression coefficients, Table S5: Model fitting results for different variable selection methods, Table S6: Weighting results calculated using the entropy method, Table S7: Positive and negative ideal solutions, Table S8: Results of TOPSIS, Figure S1: Evolutionary curves of average fitness for NSGA-III, Figure S2: Evolutionary curves of maximum fitness for NSGA-III, Figure S3: Evolutionary curves of average fitness for NSWOA, Figure S4: Evolutionary curves of maximum fitness for NSWOA. A detailed introduction to the methods section is provided in the supplementary materials [54,55,56].
