# Supplementary material for: A Systematic Intelligent Optimization Framework for a Sustained-Release Formulation Design
Source: Pharmaceutics. 2025 Nov 1;17(11):1419. doi: 10.3390/pharmaceutics17111419 (PMC12654971; doi:10.3390/pharmaceutics17111419)
Supplement: Supplementary file 1 [file pharmaceutics-17-01419-s001.zip › pharmaceutics-3919366-supplementary.pdf]

A Systematic Intelligent Optimization Framework for Sustained-Release Formulation Design

1. LASSO

Least Absolute Shrinkage and Selection Operator (LASSO) is a regularization-based variable selection and parameter estimation method proposed by Tibshirani in 1996. Its core principle involves introducing L1 paradigm penalty terms into the loss function of traditional least squares regression, achieving variable selection by shrinking regression coefficients[13]. Its optimization form can be expressed as:

$$min_{\beta}\left(\frac{1}{n}(Y-X\beta)^{T}(Y-X\beta)+\lambda\sum_{k=1}^{p}|\beta_k|^{\gamma}\right) \tag{1}$$

Where, λ is the penalty coefficient, and $\gamma > 0$ is an undetermined parameter. When γ equals 1, it corresponds to the Lasso model.

The advantage of LASSO lies in its ability to simultaneously perform parameter estimation and variable selection within the same framework. This approach avoids the high computational complexity and unstable results associated with traditional subset selection methods, while also exhibiting greater sparsity than ridge regression. Consequently, it is particularly well-suited for high-dimensional scenarios or situations where $p > n$[12]. Additionally, the schemes obtained from LASSO exhibit continuity and uniqueness, facilitating result interpretation and model generalization. However, LASSO also has certain limitations: it applies equal levels of shrinkage to all regression coefficients, leading to "group selection bias" when strongly correlated variables are present—that is, a tendency to randomly retain one variable over another rather than retaining both simultaneously. Furthermore, the choice of penalty strength is highly dependent on cross-validation; improper parameter tuning may compromise model stability and predictive performance. To address these issues, researchers have proposed a series of improvement methods, such as Smoothly Clipped Absolute Deviation (SCAD) and Minimax Concave Penalty (MCP), to enhance the accuracy and stability of variable selection[14]. In summary, LASSO, as a classic regularization method, has become an essential tool in high-dimensional data analysis and variable selection research, finding extensive applications across multiple fields including pharmaceutical formulation modeling, gene screening[54], and financial risk prediction[55].

2. SCAD

SCAD was proposed by Fan and Li in 2001 to address some limitations of LASSO in variable selection. Its core idea involves constructing a non-convex penalty function that strongly compresses small coefficients for variable screening while imposing only limited shrinkage on larger coefficients, thereby avoiding estimation bias. The penalty function is defined as:

$$p(\beta;\lambda)=\lambda\int_{0}^{\beta} min\{1,\frac{\left(\gamma-\frac{x}{\lambda}\right)_{+}}{\gamma-1}\}dx,\beta\geq 0,\gamma>2 \tag{2}$$

where $\lambda > 0$ is the penalty parameter and $a > 2$ is the tuning parameter, typically set to 3.7. This design ensures that when the coefficients are large, the SCAD penalty approaches zero, thereby making its estimator unbiased.

The primary advantages of the SCAD method include: ① It possesses Oracle-like properties, meaning it converges probabilistically to the true model when the sample size is sufficiently large, demonstrating consistent variable selection capabilities; ② It exhibits greater stability in retaining important variables, effectively avoiding the over-shrinkage issues associated with LASSO; ③ When strongly correlated variables are present, its selection results are more reasonable, reducing instances of random elimination[15]. However, SCAD also has certain limitations: due to the non-convex form of the penalty function, the optimization process is prone to getting stuck in local optima, and its computational results depend on the initial value setting and the choice of optimization algorithm. Additionally, its computational complexity is higher than LASSO, making it unsuitable for extremely high-dimensional or very large-scale data scenarios. Overall, SCAD balances sparsity and unbiasedness in variable selection and parameter estimation, making it particularly suitable for scenarios requiring precise identification of key variables. It demonstrates significant application potential in fields such as pharmaceutical formulation modeling, epidemiological research, and gene expression data analysis[15].

3. MCP

MCP was proposed by Zhang in 2010 as an improved non-convex regularization method designed to further reduce estimation bias while preserving sparsity. Its penalty function is defined as:

$$p(\beta;\lambda) = \lambda \int_0^{\beta} \left[1 - \frac{x}{(\gamma\lambda)_+}\right] dx \quad (3)$$

where $\lambda > 0$ is the penalty parameter and $\gamma > 1$ is the concavity adjustment parameter. Similar to SCAD, MCP applies strong shrinkage in the small coefficient range while gradually reducing the penalty in the large coefficient range until no constraint is imposed, thereby mitigating large coefficient bias. The advantages of MCP are primarily reflected in the following aspects: ① By controlling the penalty strength through the concavity parameter γ, it achieves a good balance between variable selection sparsity and unbiased parameter estimation; ② Compared to SCAD, its penalty function is more concise, computationally efficient, and converges more readily during optimization; ③ Under non-saturated data conditions (p>n), MCP significantly reduces the risk of local optima, demonstrating superior robustness. However, a limitation of MCP lies in its performance being dependent on the proper tuning of the concavity parameter γ. Optimal results may require multiple rounds of parameter tuning across different datasets[14]. Furthermore, while MCP mitigates the issue of local optima, variable selection instability may still occur in scenarios involving highly correlated variables in high-dimensional settings. In summary, as a rapidly evolving regularization method in recent years, MCP has gained widespread application in high-dimensional statistical modeling, machine learning, and pharmaceutical formulation optimization due to its balanced advantages of sparsity, unbiasedness, and computational efficiency[25]. It demonstrates strong applicability when addressing non-saturated data issues.

4. QIF

The Quadratic Inference Function (QIF) was introduced by Qu et al. in 2000 as an extension and refinement of GEE[26]. This method is primarily used for handling data with repeated measurements or longitudinal correlation characteristics, and is particularly well-suited for modeling problems under complex correlation structures. Unlike GEE, which relies on the precise specification of the working correlation matrix, QIF approximates the inverse of the correlation matrix through a linear combination of basis matrices, thereby enabling efficient inference without directly estimating complex correlation parameters[18]. The fundamental concept involves assuming that the response variable Y_{it} originates from repeated measures data within the generalized linear model framework, where certain correlations exist among observations. QIF transforms the inference problem into the following quadratic minimization:

$$Q_n(\beta) = g_n(\beta)^T C_n(\beta)^{-1} g_n(\beta) \tag{4}$$

where $g_n(\beta)$ denotes the extended estimation equation constructed based on a set of orthogonal basis matrices, and $C_n(\beta)$ represents its estimated covariance matrix. Parameter estimates are obtained by minimizing $Q_n(\beta)$. By avoiding explicit estimation of correlated parameters, QIF demonstrates higher robustness and efficiency under finite sample conditions. Compared to GEE, QIF offers the following advantages: ① It maintains consistency even when the working-related matrix is inaccurately specified, effectively reducing the risk of model specification bias; ② Through quadratic construction, it naturally incorporates model goodness-of-fit tests, facilitating model comparison and evaluation; ③ Under conditions of complex related structures or limited sample sizes, QIF often outperforms GEE in efficiency. However, QIF also has certain limitations: its estimation relies on the choice of basis matrices, and improper selection may compromise estimation efficiency. Additionally, under high-dimensional or extremely large-scale data conditions, covariance matrix estimation and its inverse matrix operations may still impose computational burdens.

In sustained-release formulation research, cumulative drug release rates often yield longitudinally correlated data through repeated measurements over time. Traditional methods struggle to adequately capture the dependency structure between time points. The introduction of QIF enables better handling of repeated measurement characteristics in modeling, enhancing model fitting accuracy and robustness, thereby providing more reliable statistical support for formulation optimization.

5. Exterior Penalty Function Method

In multi-objective optimization problems, besides simultaneously handling multiple conflicting objective functions, various constraints often accompany them. In the formulation optimization of sustained-release preparations through mixture design, the sum of the component contents must equal 1 (or 100%)[56, 27]. Such constant-sum constraint represents a typical example of equality constraints. To address such constrained optimization problems, the exterior penalty function method is commonly employed. The fundamental concept of this approach involves introducing a penalty term into the objective function, thereby transforming the original constrained optimization problem into an unconstrained one for scheme. The key lies in the selection and design of the penalty factor[14, 25, 29].

For general equality-constrained optimization problems:

$$minf(x), \quad s.t. hj(x) = 0, \quad j = 1,2, \ldots, l \tag{5}$$

$f(x)$ and $hj(x)$ are both continuous functions. The exterior penalty function method constructs the following auxiliary function:

$$F_1(x,\sigma) = f(x) + \sigma \sum_{j=1}^{l} h_j^2(x) \tag{6}$$

Where $\sigma > 0$ is the penalty factor, typically chosen as a large positive number. As σ increases, schemes violating the constraints incur greater penalties, causing the optimization process to progressively converge toward the feasible solution space of the original constrained problem. This transforms the original constrained optimization problem into an unconstrained one:

$$minF_1(x,\sigma) \tag{7}$$

For more general cases, the penalty function can be defined as:

$$F(x,\sigma) = f(x) + \sigma P(x) \tag{8}$$

Among which

$$P(x) = \sum_{j=1}^{l} \varphi\left(h_j(x)\right) \tag{9}$$

$\varphi(\cdot)$ is a non-negative function used to characterize the penalty for constraint violations. Through the above method, complex constrained optimization problems can be simplified into unconstrained problems, thereby facilitating their scheme.

6. NSGA-Ⅲ

NSGA-III is an improved multi-objective evolutionary algorithm proposed by Deb et al. in 2014, representing a significant extension of NSGA-II. Unlike NSGA-II, which primarily relies on the crowding distance to maintain population diversity, NSGA-III incorporates a reference point mechanism and adaptive normalization methods, enabling superior performance in handling high-dimensional multi-objective optimization problems. The fundamental concept of the algorithm is based on a rapid non-dominated sorting strategy, combined with uniformly distributed reference points to select individuals. This ensures a more even distribution of Pareto frontier solutions across the objective space[30, 31]. The main steps include: first, initializing the population and reference points while setting parameters such as crossover and mutation probabilities; subsequently, generating offspring populations through crossover and mutation, merging them with the parent generation, and retaining optimal individuals via non-dominated sorting; then calculating ideal points and normalizing the solution set by associating individuals with their nearest reference points to ensure uniformity. Through this mechanism, NSGA-III effectively enhances convergence speed and global optimization capability while preserving population

diversity, making it particularly suitable for complex optimization problems with high-dimensional objectives.

7. MOGWO

MOGWO is a multi-objective version of the Grey Wolf Optimization (GWO) algorithm proposed by Mirjalili et al. in 2016. This algorithm simulates the social hierarchy and group predatory behavior of grey wolves, guiding the search for optimal solutions through three types of “leader wolves” α, β, and δ thereby balancing global exploration and local exploitation capabilities. Unlike single-objective GWO, MOGWO incorporates an external archive to preserve non-dominated solutions and employs non-domination sorting with a crowding distance mechanism to filter the solution set. During each iteration, the algorithm progressively approaches the Pareto front by updating the wolf pack's positions, while leveraging randomness to enhance search diversity and prevent getting stuck in local optima. The Archive mechanism not only ensures the preservation of historically optimal solutions but also selects new leading wolves in less congested regions through a roulette wheel approach, thereby maintaining the uniformity and distribution of the solution set[32, 33]. Overall, MOGWO enhances algorithmic convergence speed while generating more representative Pareto solution sets, making it suitable for complex multi-objective optimization scenarios.

8. NSWOA

Whale Optimization Algorithm (WOA) is an intelligent optimization algorithm inspired by the hunting behavior of humpback whales, proposed by Mirjalili and Lewis in 2016. Its core mechanism incorporates three strategies: encircling prey, spiral bubble net feeding, and random search. It features a simple structure, minimal parameters, and strong robustness[34]. Non-dominated Sorting Whale Optimization Algorithm (NSWOA) is an improved algorithm proposed by Pradeep and Narottam in 2017 based on WOA, specifically designed for solving multi-objective optimization problems. This method incorporates non-dominated sorting and crowding distance into the whale algorithm framework to hierarchically organize and filter the solution set. Non-dominated sorting ensures the correct construction of the Pareto front, while crowding distance maintains solution diversity by measuring the distribution density among solutions[35]. This enables the population to cover the Pareto front more uniformly within the objective space. Compared to traditional WOA, NSWOA not only retains the advantages of WOA in global search and strong local development capabilities but also achieves superior results in solution set distribution and global equilibrium. Consequently, it demonstrates greater adaptability and stability in multi-objective optimization problems.

9. EWM

Entropy Weight Method (EWM) is an objective weight determination technique based on information entropy theory. It measures the information entropy of each evaluation indicator to reflect the information content of the indicator, thereby determining its weight. This method eliminates subjectivity, making the evaluation results more objective and reliable[37]. The steps of the entropy weight method are as follows: First, construct the decision matrix X for Pareto optimal solutions, where the indicator values for each alternative undergo dimensionless processing. For positive indicators, the following normalization formula is applied:

$$y_{ij} = \frac{x_{ij} - x_j^{min}}{x_j^{max} - x_j^{min}} \tag{10}$$

The standardization formula for negative indicators is:

$$y_{ij} = \frac{x_j^{max} - x_{ij}}{x_j^{max} - x_j^{min}} \tag{11}$$

Then, calculate the weight P_{ij} and entropy value e_j for each indicator, and determine the objective weight w_j for each indicator based on the entropy value:

$$w_j = \frac{g_j}{\sum_{j=1}^{m} g_j} \tag{12}$$

$g_j = 1 - e_j$, indicating the degree of divergence in the indicators.

10. TOPSIS

TOPSIS is a commonly used multi-alternative decision-making method that evaluates the relative merits of alternatives by calculating their distance from an ideal solution[38]. First, the standardized data obtained from the entropy weight method undergoes weighting to yield the weighted evaluation matrix R. Next, the positive and negative ideal solutions S_j+ and S_j– are determined, where the positive ideal solution corresponds to all maximum values and the negative ideal solution corresponds to all minimum values:

$$S_j^{+} = \max_{1 \le i \le m} r_{ij} \tag{13}$$

$$S_j^{-} = \min_{1 \le i \le m} r_{ij} \tag{14}$$

Then, compute the weighted euclidean distance between each solution and the positive and negative ideal solutions:

$$Sd_i^{+} = \sqrt{\sum_{j=1}^{n} \left(S_j^{+} - r_{ij}\right)^2}, i = 1,2,\cdots,m \tag{15}$$

$$Sd_i^{-} = \sqrt{\sum_{j=1}^{n} \left(S_j^{-} - r_{ij}\right)^2}, i = 1,2,\cdots,m \tag{16}$$

By calculating the relative proximity η_i of each solution to the ideal solution:

$$\eta_i = Sd^- / (Sd_i^+ + Sd_i^-), i = 1,2,\cdots,m \tag{17}$$

Finally, the Pareto optimal solutions are ranked in descending order based on relative closeness, and the optimal solution η_i' is selected.

11. Prescription Components and Dosage Ranges

Table S1 Scope setting table for each component

Components	Scope w/%	
	Lower limit	Upper limit
X_1	25	47.8
X_2	10	27.8
X_3	2	10
X_4	15	22.8
X_5	5	12.8

Note: The combined proportion of glipizide inclusion complex and magnesium stearate is 17.2%. The sum of X_1, X_2, X_3, X_4, and X_5 must equal 82.8.

12. Feature variables selected by Lasso, SCAD, and MCP variable selection methods and their regression coefficients

Table S2 Lasso screening variables and regression coefficients

$\hat{\beta}_1$	$\hat{\beta}_4$	$\hat{\beta}_{23}$	$\hat{\beta}_{25}$	$\hat{\beta}_{134}$	$\hat{\beta}_{35}$
0.1765	-0.1098	0.02962	-0.01353	-0.0555	-0.02336
$\hat{\beta}_{123}$	$\hat{\beta}_{1245}$	$\hat{\beta}_{12345}$	$\hat{\beta}_{1t}$	$\hat{\beta}_{2t}$	$\hat{\beta}_{3t}$
0.0002387	0.00004199	0.00009892	0.00697	0.000000000003365	0.03215
$\hat{\beta}_{4t}$	$\hat{\beta}_{5t}$	$\hat{\beta}_{t^2}$	$\hat{\beta}_t$		
0.0266	-0.0002094	-0.2097	7.994		

Table S3 SCAD screening variables and regression coefficients

$\hat{\beta}_4$	$\hat{\beta}_{24}$	$\hat{\beta}_{25}$	$\hat{\beta}_{35}$	$\hat{\beta}_{45}$	$\hat{\beta}_{134}$
-0.00000006468	-0.00110	-0.01517	-0.01043	0.00915	-0.0001215
$\hat{\beta}_{12345}$	$\hat{\beta}_{2t}$	$\hat{\beta}_{3t}$	$\hat{\beta}_{5t}$	$\hat{\beta}_{t^2}$	$\hat{\beta}_t$
0.00002195	-0.0004406	0.02336	-0.0005657	-0.2194	9.096

Table S4 MCP screening variables and regression coefficients

$\hat{\beta}_{24}$	$\hat{\beta}_{25}$	$\hat{\beta}_{35}$	$\hat{\beta}_{134}$	$\hat{\beta}_{135}$	$\hat{\beta}_{12345}$
-0.00555	-0.02004	-0.00576	-0.0014	-0.0001077	0.00001217

$\hat{\beta}_{3t}$	$\hat{\beta}_{5t}$	$\hat{\beta}_{t^2}$	$\hat{\beta}_t$
0.02594	-0.0000693	-0.2234	9.084

13. Expressions of sub-objective functions

When t = 2 h, the sub-objective function was:

$$\hat{Y}_2 = 13.02228 - 0.01113X_2X_4 - 0.00974X_2X_5 - 0.61399X_3X_5 - 0.00165X_1X_3X_4 + 0.01076X_1X_3X_5 + 0.00005X_1X_2X_3X_4X_5 + 0.04946X_3 - 0.00098X_5$$

When t = 8 h, the sub-objective function was:

$$\hat{Y}_8 = 52.08912 - 0.01113X_2X_4 - 0.00974X_2X_5 - 0.61399X_3X_5 - 0.00165X_1X_3X_4 + 0.01076X_1X_3X_5 + 0.00005X_1X_2X_3X_4X_5 + 0.19784X_3 - 0.00392X_5$$

When t = 24 h, the sub-objective function was:

$$\hat{Y}_{24} = 156.26736 - 0.01113\mathrm{X}_2\mathrm{X}_4 - 0.00974\mathrm{X}_2\mathrm{X}_5 - 0.61399\mathrm{X}_3\mathrm{X}_5 - 0.00165\mathrm{X}_1\mathrm{X}_3\mathrm{X}_4 + 0.01076\mathrm{X}_1\mathrm{X}_3\mathrm{X}_5 + 0.00005\mathrm{X}_1\mathrm{X}_2\mathrm{X}_3\mathrm{X}_4\mathrm{X}_5 + 0.59352\mathrm{X}_3 - 0.01176\mathrm{X}_5$$

14. Model Fitting Results for Different Variable Selection Methods

Table S5 Model fitting results for different variable selection methods

Variable selection methods	Model evaluation metrics	
	AIC	BIC
All characteristic variables + QIF	43.8028	97.1050
Lasso + QIF	31.8028	71.2001
SCAD + QIF	23.8028	53.9301
MCP + QIF	19.8028	45.2951

15. The average fitness and maximum fitness evolution curves of NSGA-III and NSWOA

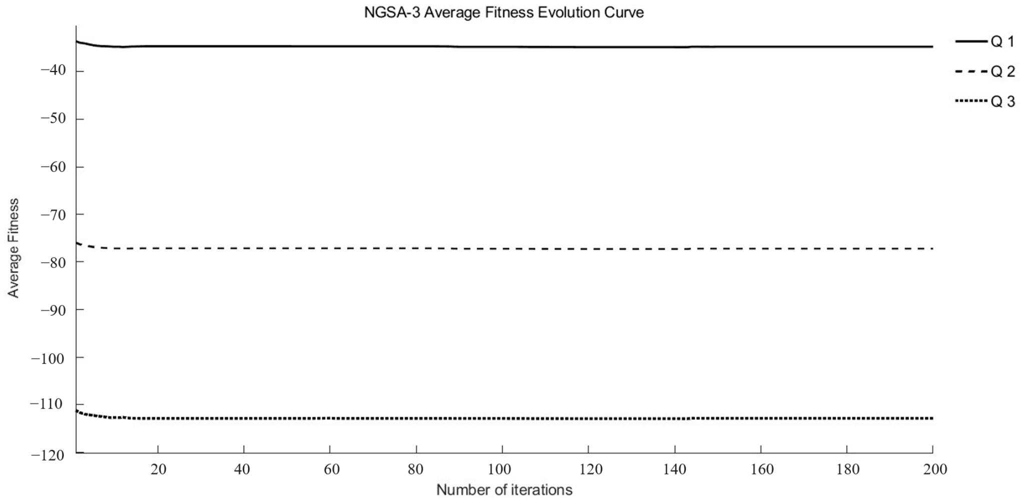

Figure S1 Evolutionary curves of average fitness for NSGA-III

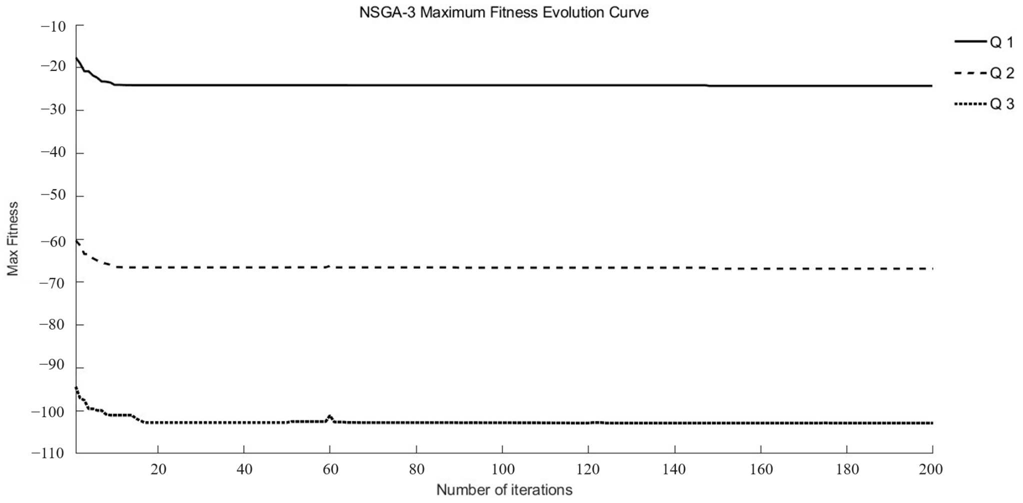

Figure S2 Evolutionary curves of maximum fitness for NSGA-III

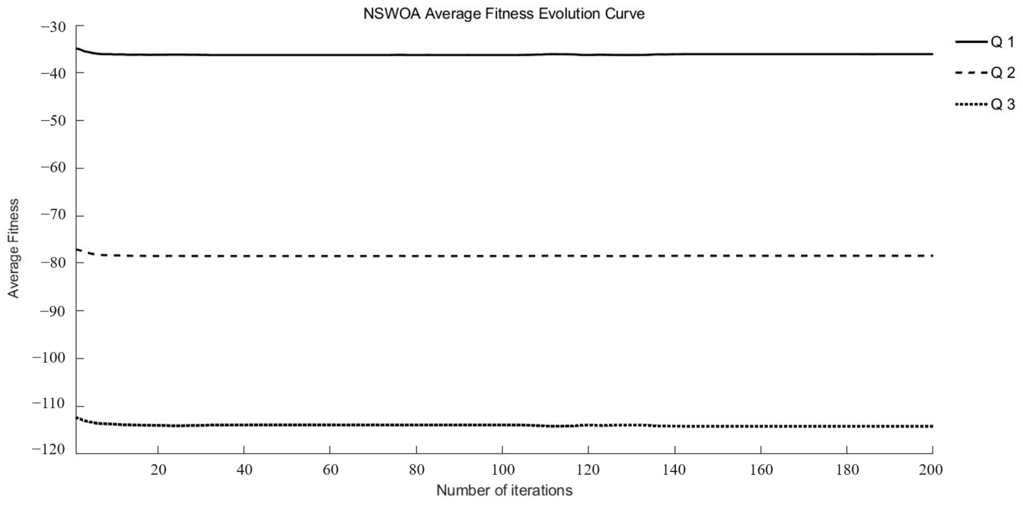

Figure S3 Evolutionary curves of average fitness for NSWOA

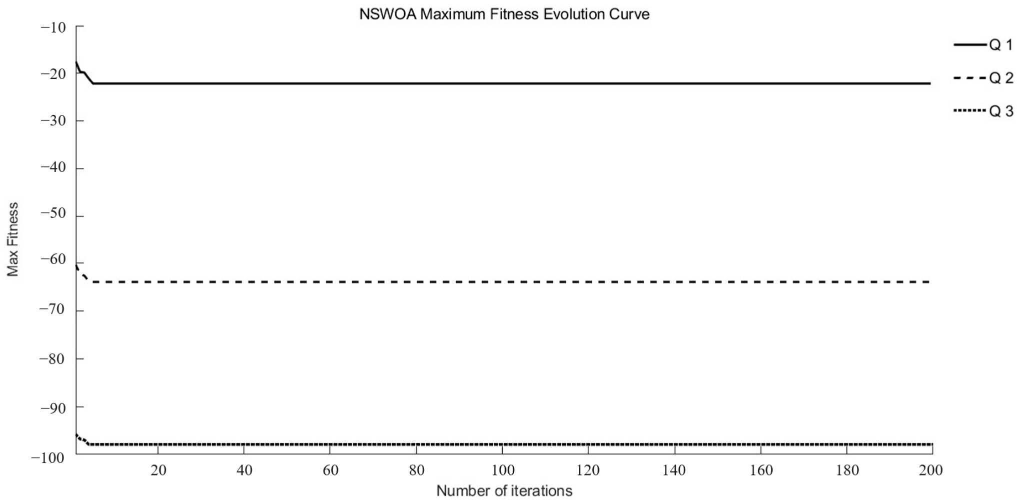

Figure S4 Evolutionary curves of maximum fitness for NSWOA

16. Weight calculation results using the entropy value method

Table S6 Weighting results calculated using the entropy method

Cumulative release degree	Information entropy value e	Information utility value d	Weighting coefficient w
Y_2	0.9750	0.0250	27.50%
Y_8	0.9668	0.0332	36.47%
Y_{24}	0.9672	0.0328	36.04%

17. Positive and negative ideal solutions obtained by TOPSIS

Table S7 Positive and negative ideal solutions

Cumulative release degree	Optimal ideal solution A+	Negative ideal solution A-
Y_2	0.062	0.000
Y_8	0.084	0.000
Y_{24}	0.089	0.000

18. Results of the TOPSIS

Table S8 Results of TOPSIS

Schemes	Optimal ideal solution distance D+	Negative ideal solution distance D-	Relative proximity C	Sorting results
45	0.009	0.129	0.932	1

29	0.013	0.128	0.908	2
15	0.014	0.123	0.901	3
44	0.014	0.125	0.898	4
43	0.022	0.117	0.839	5
40	0.023	0.115	0.834	6
42	0.023	0.115	0.831	7
38	0.024	0.113	0.823	8
41	0.025	0.114	0.819	9
30	0.028	0.118	0.809	10
39	0.029	0.109	0.79	11
26	0.03	0.109	0.782	12
28	0.035	0.102	0.747	13
14	0.038	0.101	0.728	14
37	0.038	0.098	0.72	15
36	0.042	0.095	0.695	16
11	0.042	0.097	0.695	17
12	0.045	0.094	0.675	18
27	0.046	0.09	0.662	19
13	0.051	0.092	0.644	20
34	0.049	0.088	0.642	21
35	0.049	0.088	0.642	22
8	0.057	0.087	0.605	23
33	0.056	0.081	0.589	24
20	0.059	0.082	0.581	25
32	0.058	0.079	0.577	26
10	0.059	0.079	0.571	27
7	0.064	0.074	0.536	28
9	0.065	0.071	0.522	29
6	0.071	0.065	0.476	30
25	0.075	0.066	0.467	31
24	0.075	0.065	0.464	32
31	0.08	0.059	0.426	33
23	0.089	0.055	0.381	34
22	0.09	0.054	0.376	35
21	0.088	0.052	0.369	36
17	0.096	0.053	0.356	37
16	0.103	0.048	0.317	38

3	0.097	0.043	0.307	39
19	0.106	0.036	0.252	40
5	0.109	0.034	0.236	41
4	0.107	0.032	0.232	42
1	0.117	0.035	0.23	43
18	0.11	0.03	0.216	44
2	0.126	0.017	0.119	45

References

[13]. Tibshirani R, Bien J, Friedman J, Hastie T, Simon N, Taylor J, Tibshirani RJ. Strong rules for discarding predictors in lasso-type problems[J]. Journal of the Royal Statistical Society Series B, Statistical methodology. 2012,74(2):245-66.

12]. Simon N, Friedman J, Hastie T, Tibshirani R. Regularization Paths for Cox's Proportional Hazards Model via Coordinate Descent[J]. Journal of statistical software. 2011,39(5):1-13.

[14]. Fan J, Lv J. Non-Concave Penalized Likelihood with NP-Dimensionality[J]. IEEE transactions on information theory. 2011,57(8):5467-84.

[54]. Witten DM, Tibshirani RJ. Extensions of sparse canonical correlation analysis with applications to genomic data[J]. Statistical applications in genetics and molecular biology. 2009,8(1):Article28.

[55]. Friedman J, Hastie T, Tibshirani R. Regularization Paths for Generalized Linear Models via Coordinate Descent[J]. Journal of statistical software. 2010,33(1):1-22.

[15]. Breheny P, Huang J. COORDINATE DESCENT ALGORITHMS FOR NONCONVEX PENALIZED REGRESSION, WITH APPLICATIONS TO BIOLOGICAL FEATURE SELECTION[J]. The annals of applied statistics. 2011,5(1):232-53.

[25]. Huang J, Breheny P, Ma S. A Selective Review of Group Selection in High-Dimensional Models[J]. Statistical science : a review journal of the Institute of Mathematical Statistics. 2012,27(4).

[26]. Westgate PM. Criterion for the simultaneous selection of a working correlation structure and either generalized estimating equations or the quadratic inference function approach[J]. Biometrical journal Biometrische Zeitschrift. 2014,56(3):461-76.

[18]. Odueyungbo A, Browne D, Akhtar-Danesh N, Thabane L. Comparison of generalized estimating equations and quadratic inference functions using data from the National Longitudinal Survey of Children and Youth (NLSCY) database[J]. BMC medical research methodology. 2008,8:28.

[56]. Habib BA, Abdeltawab NF, Salah Ad-Din I. D-optimal mixture design for optimization of topical dapsone niosomes: in vitro characterization and in vivo activity against Cutibacterium acnes[J]. Drug delivery. 2022,29(1):821-36.

[27]. Hare LB, Altan S, Coppenolle H. Correction to "Mixture Experimentation in Pharmaceutical Formulations: A Tutorial"[J]. Pharmaceutical statistics. 2025,24(4):e70017.

[29]. Wang L, Kim Y, Li R. CALIBRATING NON-CONVEX PENALIZED REGRESSION IN ULTRA-HIGH DIMENSION[J]. Annals of statistics. 2013,41(5):2505-36.

[30]. Lyu J, Jiang Y, Xu C, Liu Y, Su Z, Liu J, He J. Multi-objective winter wheat irrigation strategies optimization based on coupling AquaCrop-OSPy and NSGA-III: A case study in Yangling, China[J]. Sci Total Environ. 2022,843:157104.

[31]. Wang S, Wang Y, Wang Y, Wang Z. Comparison of multi-objective evolutionary algorithms applied to watershed management problem[J]. Journal of environmental management. 2022,324:116255.

[32]. Saremi, Shahrzad, Coelho, Leandro, dos, S., Mirjalili, Seyed, Mohammad, Seyedali. Multi-objective grey wolf optimizer: A novel algorithm for multi-criterion optimization[J]. Expert Systems with Application. 2016,47(Apr.1):106-19.

[33]. Al-Qaness MAA, Helmi AM, Dahou A, Elaziz MA. The Applications of Metaheuristics for Human Activity Recognition and Fall Detection Using Wearable Sensors: A Comprehensive Analysis[J]. Biosensors. 2022,12(10).

[34]. Nadimi-Shahraki MH, Zamani H, Asghari Varzaneh Z, Mirjalili S. A Systematic Review of the Whale Optimization Algorithm: Theoretical Foundation, Improvements, and Hybridizations[J]. Archives of computational methods in engineering : state of the art reviews. 2023:1-47.

[35]. Jangir P, Jangir N. Non-Dominated Sorting Whale Optimization Algorithm (NSWOA): A Multi-Objective Optimization Algorithm for Solving Engineering Design Problems[J]. 2017.

[37]. Pan X, Kang Y, Lu JF, Yu XZ. Using the TOPSIS method to select the best low-toxicity organic cosolvent for rice-based toxicity tests[J]. Ecotoxicol Environ Saf. 2025,290:117733.

[38]. de Oliveira BR, Zuffo AM, Aguilera JG, Steiner F, Ancca SM, Flores LAP, Gonzales HHS. Selection of Soybean Genotypes under Drought and Saline Stress Conditions Using Manhattan Distance and TOPSIS[J]. Plants (Basel, Switzerland). 2022,11(21).
